# Transcriptome profiling reveals Silibinin dose-dependent response network in non-small lung cancer cells

**DOI:** 10.7717/peerj.10373

**Published:** 2020-12-16

**Authors:** Jagan Mohan Kaipa, Vytaute Starkuviene, Holger Erfle, Roland Eils, Evgeny Gladilin

**Affiliations:** 1 Helmholtz Center for Infection Research, Braunschweig, Germany; 2 BioQuant, University Heidelberg, Heidelberg, Germany; 3 Theoretical Bioinformatics, German Cancer Research Center, Heidelberg, Germany; 4 Institute of Biosciences, Vilnius University Life Science Center, Vilnius, Lithuania; 5 Center for Digital Health, Berlin Institute of Health and Charité Universitätsmedizin Berlin, Berlin, Germany; 6 Health Data Science Unit, Heidelberg University Hospital, Heidelberg, Germany; 7 Leibniz Institute of Plant Genetics and Crop Plant Research, Seeland, Germany; 8 Applied Bioinformatics, German Cancer Research Center, Heidelberg, Germany

**Keywords:** Silibinin, Custom drug response, Adjuvant cancer therapy, Transcriptome profiling, Drug susceptibility network

## Abstract

Silibinin (SIL), a natural flavonolignan from the milk thistle (Silybum marianum), is known to exhibit remarkable hepatoprotective, antineoplastic and EMT inhibiting effects in different cancer cells by targeting multiple molecular targets and pathways. However, the predominant majority of previous studies investigated effects of this phytocompound in a one particular cell line. Here, we carry out a systematic analysis of dose-dependent viability response to SIL in five non-small cell lung cancer (NSCLC) lines that gradually differ with respect to their intrinsic EMT stage. By correlating gene expression profiles of NSCLC cell lines with the pattern of their SIL IC50 response, a group of cell cycle, survival and stress responsive genes, including some prominent targets of STAT3 (BIRC5, FOXM1, BRCA1), was identified. The relevancy of these computationally selected genes to SIL viability response of NSCLC cells was confirmed by the transient knockdown test. In contrast to other EMT-inhibiting compounds, no correlation between the SIL IC50 and the intrinsic EMT stage of NSCLC cells was observed. Our experimental results show that SIL viability response of differently constituted NSCLC cells is linked to a subnetwork of tightly interconnected genes whose transcriptomic pattern can be used as a benchmark for assessment of individual SIL sensitivity instead of the conventional EMT signature. Insights gained in this study pave the way for optimization of customized adjuvant therapy of malignancies using Silibinin.

## Introduction

The ability of malignant cells to attain drug resistance and to escape cell death frequently observed in different types of cancer represents the major challenge to chemical tumor therapy. Several evolutionary conserved mechanisms mediate elevated survival capabilities and drug resistance of cancer cells including inhibition of apoptotic pathways, alteration and enhancement of metabolism, DNA damage repair, drug inactivation or alteration, genetic and epigenetic activation of stress response and proliferation programs such as epithelial-mesenchymal transition (EMT) (Michael & Doherty, 2005; Hang, Cai & Fan, 2013; Housman et al., 2014; Hanahan & Weinberg, 2011). As a consequence of elevated environmental adaptation and self-reprogramming capabilities, cancer cells often attain resistance against targeted drugs and even drug combinations by bypassing affected pathways (Patel & Rothenberg, 1994; Clarke et al., 2019). In most of the tumors, multiple survival mechanisms and pathways are active in parallel. A network of dozens tightly interconnected genes rather than just few linear signaling pathways maintain abnormal survival and resistance capabilities of cancer cells  (Gladilin & Eils, 2017).

A promising approach to overcome the limitations of conventional therapy is a combination of targeted and sensitizing drugs (Chandarlapaty et al., 2010; Al-Lazikani, Banerji & Workman, 2012). In view of often unavoidable side effects of targeted therapy, sensitizing compounds with a low toxicity for healthy cells are preferred that are still capable to weaken abnormally functioning tumor cells. There is an increasing body of evidence that secondary plant metabolytes such as various polyphenolic compounds exhibit distinctive antineoplastic properties that make them promising sensitizing agents for combined tumor therapy (Gerhäuser, 2012; Wang et al., 2016; Dayem et al., 2016; Thomford et al., 2018). Some of these natural compounds have a long history of usage as nutrition supplements in traditional medicine, and were empirically proven to be well tolerated.

One of the antineoplastic phytocompounds increasingly gaining attention in the last two decades is Silibinin (also known as Silybin or Silymarin)—a flavonolignan from the milk thistle *Silybum marianum*. Silibinin is a mixture of two diastereoisomers Silybin A and Silybin B, at a ratio of 1:1 (Agarwal et al., 2003). Originally known as a hepatoprotective dietary drug (Machicao & Sonnenbichler, 1977; Sonnenbichler et al., 1999; Flora et al., 1998; Vargas-Mendoza, 2014; Hellerbrand et al., 2016), in recent years Silibinin has been shown to exhibit remarkable antineoplastic properties crossover different types of tumors which was attributed to different molecular mechanisms and signaling pathways (Fraschini, Demartini & Esposti, 2002; Gazak, Walterova & Kren, 2007; Ramasamy & Agarwal, 2008; Ting, Deep & Agarwal, 2013; Polachi et al., 2016). Meanwhile more than 1,500 studies on hepatoprotective and antineoplastic effects of Silibinin and their mechanisms in different tissues and cells were published (Pubmed, 2019). The predominant majority of these works were, however, performed with one particular cell line and typically focused on a few molecular targets and signaling/metabolic pathways directly or indirectly affected by Silibinin, see Table 1. Primary molecular targets and mechanisms of SIL action in particular type of cancer tissue/cells were usually hypothesized and rarely investigated mechanistically. As direct molecular binding targets of Silibinin several drivers and mediators of malignant transformation were reported, see Table 2.

**Table 1:** Overview of studies on antineoplastic effects of Silibinin in different cancer tissues/cells.

Cancer tissue, cell type	Molecular mechanisms	Lit.
H. lymphoma, U-937	Inh: TNF, NF-kB, MAPK8	Manna et al. (1999)
H. colon cancer, HT-29	Up: p27, p21	Agarwal et al. (2003)
	Down: CCNE1, CCND1, CDC25C, CCNB1	
	Inh: CDK2, CDK4, CDC2	
M. SKH-1 epidermis	Up: p53, p21	Dhanalakshmi et al. (2004)
H. endothelium, ECV304	Up: Bax	Yoo et al. (2004)
	Down: Bcl-2	
	Inh: NF-kB	
	Act: CASP3, CASP9	
M. keratinocytes, JB6 C141	Up: p53, Bax, CYCS	Katiyar, Roy & Baliga (2005)
	Down: Bcl-2	
	Act: CASP3, APAF1, PARP-1	
H. colorectal cancer, SW480	Inh: PIK3CA-Akt-mTOR	Raina et al. (2013)
	Act: MAP2K1/2-MAPK1/3	
H. breast cancer, MCF-7	Inh: HSP90	Zhao et al. (2011)
H. lung cancer, A549	Inh: PI3K-Akt-MAPK	Chen et al. (2005)
H. breast cancer, MCF-7, MDA-MB-231	Inh: Notch-1	Kim et al. (2014b)
	Down: ERK, Akt, AIF, CASP3	
H. lung cancer	Inh: EGFR	Hou et al. (2018)
H. colorectal adenocarcinoma, LoVo	Inh: GLUT1	Catanzaro et al. (2018)
H. glioblastoma, A172, SR	Inh: mTor, Yap	Bai et al. (2018)
H. prostate cancer, DU145, PC3	Up: p27, p21	Davis-Searles et al. (2005)
		Deep et al. (2006), Mokhtari, Motamed & Shokrgozar (2008)
	Down: CDK4, CDK6, CDK2,	
	CCNE1, CCND1, CCNB1	
H. umbilical vein endothelial cells, HUVEC	Inh: NF-kB	Kang et al. (2003)
	Down: VCAM-1, ICAM-1, CD62E	
H. lung cancer, PC-9	Up: CDH1	Cufí et al. (2013)
	Down: VIM	
H. bladder cancer, T24	Inh: CTNNB1/ZEB1	Wu et al. (2013)
H. breast cancer, MDA-MB-231	Inh: CXCR4	Wang et al. (2014a)
H. breast cancer, MDA-MB-231	Down: CDC42, D4-GDI	Dastpeyman et al. (2012)
H. leukemia, THP-1	Inh: p65, ICAM-I	Chen et al. (2014)
H. lung cancer, A549	Inh: STAT1, STAT3, NF-kB	Chittezhath et al. (2008)
H. cervical cancer, HeLa;	Inh: STAT1, STAT3, NF-kB	García-Maceira & Mateo (2009)
H. hepatoma, Hep3B		
H. breast cancer, MCF7	Up: BNIP3	Jiang et al. (2015)
H. prostate cancer, PCA	Down: SREBP1/2	Nambiar et al. (2014)
	Up: AMPK	
H. prostate cancer, LNCaP	Up: p21, p27	Zi & Agarwal (1999)
	Down: CCND1, CDK4, CDK6	
H. colon cancer	Up: Bax	Kauntz et al. (2012)
	Down: Bcl-2, IL1*β*, TNF*α*, MMP7	
H. colon cancer, SW480	Act: CASP3, CASP8	Kauntz (2012)
H. lung cancer, H1975, HCC827, A549, H460, H1299	Inh: EGFR, LOX	Hou et al. (2018)
H. lung cancer	Down: HIF1A, TNF*α*, NK-kB, STAT3	Tyagi et al. (2009)
	Up: Ang-2, Tie-2, TIMP-1, TIMP-2	
H. cervical cancer, HeLa	Up: ROS, NOS	Fan et al. (2011)
H. lung cancer, MCF-7	Down: Er *α*, mTor, ERK	Zheng et al. (2015)
H. glioma, U87, U251	Down: PI3K, FOXM1	Zhang et al. (2015)
H. breast cancer, MCF7	Down: Bcl-2, BRCA1	Pirouzpanah et al. (2015)
H. colon cancer, HCT116	Inh: CD44v6	Patel et al. (2018)
	Down: Nanog, CTNNB1, CDKN2A	
	Up: CDH1	
H. prostate cancer, DU145	Inh: STAT3, pSTAT3	Agarwal et al. (2007)
H. gastric cancer, MGC803	Inh: STAT3, pSTAT3	Wang et al. (2014b)
H. pancreatic cancer, S2-013, T3M4, HEK-293T	Inh: STAT3, pSTAT3, c-Myc	Shukla et al. (2015)
H. breast cancer, MDA-MB468, BT20	Inh: STAT3	Kim et al. (2014a)
H. breast cancer, MDA-MB-231	Inh: STAT3	Byun et al. (2017)

**Notes.**

Abbreviations Up upregulation Down downregulation Inh inhibition Act activation H human M mice

**Table 2:** Overview of reported direct molecular binding targets of Silibinin.

Protein name	Gene symbol	Lit.
Cytochrom P450 2C9	CYP2C9	Beckmann-Knopp et al. (2000), Kawaguchi-Suzuki et al. (2014)
		Bijak (2017), Por et al. (2018)
CXC-Motiv-Chemokinrezeptor 4	CXCR4	Wang et al. (2014a)
Mitogen-Activated Protein Kinase 11	MAPK11	Youn et al. (2013)
G-protein coupled receptor 12	GPR120	Chinthakunta et al. (2018)
Cyclooxygenase-2	COX-2	Malik, Manan & Kirza (2017)
Phospholipase A2	PLA2	Malik, Manan & Kirza (2017)
Aldo-Keto Reductase Family 1 Member D1	AKR1D1	Malik, Manan & Kirza (2017)
Core 1 *β*1,3-galactosyltransferase	C1GALT1	Lin et al. (2018)
*β*-catenin	CTNB1	Iftikhar & Rashid (2014)
Epidermal Growth Factor Receptor	EGFR	Hosen et al. (2016)
Heat Shock Protein 90	HSP90	Zhao et al. (2011), Riebold et al. (2015), Cuys et al. (2019)
Signal Transducers and Activators of Transcription 3	STAT3	Bosch-Barrera & Menendez (2015), Bosch-Barrera, Queralt & Menedez (2017)
		Verdura et al. (2018), Qin et al. (2019)

As a multitarget compound with a broad spectrum of antineoplastic action Silibinin combines the ability to selectively reduce viability of cancer cells in a dose dependent manner (Katiyar, Roy & Baliga, 2005; Mokhtari, Motamed & Shokrgozar, 2008; Zhan et al., 2011; Su et al., 2013; Wang et al., 2014b) with low toxicity for normal tissues even in the range of high, therapeutically relevant doses of more than 1,500 mg/day (Soleimani et al., 2019). However, the knowledge of primary targets alone does not provide yet a reliable criterion for quantitative assessment of SIL efficiency in application to a particular cancer tissue/cell. One of the global factors known to be relevant to the drug dose response is the intrinsic EMT stage of cancer cells. Accordingly, cells exhibiting mesenchymal pheno-/genotypic profiles are more drug-resistant to antineoplastic drugs than epithelial cells (Tan et al., 2014). It is, however, not known whether this rule also applies to Silibinin. Here, we address this question by a systematic analysis of dose-dependent viability response to SIL in five non-small cell lung cancer (NSCLC) lines (H1650, H1975, A549, H838, H2030) that gradually differ with respect to their intrinsic EMT stage defined by the transcriptomic signature from (Byers et al., 2013). The relationship between SIL IC50 viability response and gene expression profiles of five NSCLC cell lines was compared with a reference compound, Withaferin-A, which is known to exhibit dose-dependent EMT-inhibiting effects (Vanden Berghe et al., 2012; Vyas & Singh, 2014; Gladilin, Gonzalez & Eils, 2014). Our experimental results show that in contrast to WFA SIL does not exhibit a EMT-conform dose–response. Instead, SIL viability response of NSCLC cells turns out to correlate with the expression level of cell cycle, survival and stress responsive genes including some prominent targets of *STAT3*.

## Materials and Methods

### Cell culturing

Human lung adenocarcinoma cell lines H1650, H1975, A549, H838, H2030 were obtained from ATCC and cultured in DMEM (Dulbecco’s Modified Eagle Medium) medium supplemented with 10% fetal calf serum (FCS) and 100 U/ml penicillin G and 100 µg/ml streptomycin sulfate at 37 °C in a humidified 5%CO_2_ incubator. To ensure the ample number of cell count, cells were cultured three days prior to cell seeding. When the culture plates became confluent, NSCLC cells were detached and collected in a sterile Falcon tube with a pre-warmed medium for transfection. Cells were subjected to centrifugation (at 1000 rpm and room temperature for 5 min) and the supernatant was aspirated. The cells were resuspended in the medium for transfection. The number of cells/ml was determined by using Neubauer counting chamber and cells were diluted to get a concentration of 12000 cells/100 µl of the medium. After careful optimization of transfection efficiency, 12000 cells/well in 100 µl was found to show good transfection rate.

### Silibinin and Withaferin-A compounds

Silibinin (C_25_H_22_O_10_, mol. weight 482.44) and Withaferin-A (C_28_H_38_O _6_, mol. weight 470.60) both were purchased from Sigma-Aldrich (Germany). Both compounds were dissolved in dimethylsulfoxide (DMSO; Sigma-Aldrich) to make a stock solution. The final concentration of DMSO in the culture medium did not exceed 0.5% which has no detectable effects on cells.

### CellTiter-Blue cell viability assay

Cells seeded into 96-well plates in sextuplicate at a density of 15 × 10^3^ cells per well. Twenty-four hours after seeding, the cells were treated with various concentrations of Withaferin-A and Silibinin for totally 24 h. To measure the viability of cells, CellTiter-Blue^^®^^ Viability Assay (Promega, G8081) was applied according to the manufacturer’s instructions. Incubation with the dye for 60 min was followed by measurement of the fluorescence with the infinite F200 pro Reader (TECAN). A blank well without cells was measured to determine the background. After subtraction of the background, cell dilution series enabled due to a direct correlation of the signal intensities with the cell number, the verification of absolute cell numbers.

### Determination of IC50 from CTB measurements

The half maximal inhibitory concentration (IC50) was determined from series of dose-dependent CTB measurements using following basic steps. First, the raw CTB intensity measurements (*I*_*i*_) were normalized by the intensity level (*I*_0_) of the reference (untreated) probe:

(1) \begin{eqnarray*}n{I}_{i}=100 \frac{{I}_{i}}{{I}_{0}} .\end{eqnarray*}

Subsequently, the average pattern of normalized dose response (*anI*_*i*_) from *N* different measurements was calculated:

(2) \begin{eqnarray*}an{I}_{i}= \frac{1}{N} \sum _{j=1..N}n{I}_{i}(j).\end{eqnarray*}

Finally, the IC50 value of a particular cell line and measurement condition (e.g., duration of treatment) was determined from the average normalized pattern of IC50 dose response by fitting the Hill’s sigmoid function (Spiess & Neumeyer, 2010; Sebaugh, 2011)

(3) \begin{eqnarray*}anI(D)=\min \nolimits + \frac{\max \nolimits -\min \nolimits }{1+( \frac{D}{IC50} )^{H}} ,\end{eqnarray*}

where *D* is the drug dose, min and max are the minimum and maximum values of *anI*, *IC*50 and *H* are the IC50 and the Hill’s coefficient values that are determined from the fit. The nonlinear least square fit of the Hill’s equations was performed automatically using the MATLAB R2019b (The Mathworks, Inc.). To characterize relative differences in viability response (*V*_*i*_) among five NSCLC cell lines (*i* = 1..5), normalized IC50 values (*nV*_*i*_)

(4) \begin{eqnarray*}n{V}_{i}= \frac{{V}_{i}-{V}_{\mathrm{min}}}{{V}_{\mathrm{max}}-{V}_{\mathrm{min}}} \in [0,1]\end{eqnarray*}

and their ranks (*R*_*i*_)

(5) \begin{eqnarray*}{R}_{i}=\text{rank}({V}_{i},[{V}_{1},{V}_{2},{V}_{3},{V}_{3},{V}_{4},{V}_{5}]).\end{eqnarray*}

were calculated.

### Determination of total cell count using quantitative image analysis

Quantitative image analysis was performed to acquire the total cell count. Immediately after the measurement of the fluorescence signal, the medium is aspirated from 96well plate containing NSCLC cells and washed with PBS solution twice to ensure the complete removal of dead cells. Since dead cells do not attach to the surface and freely float in the medium, washing twice with PBS removes approximately all the dead cells from the well. After washing, 70 µl cell fixation solution (PFA 3% + Hoechst dye) was added to each well for the fixation and nuclei staining of cells by a single step. Cells were incubated at 4 °C for 48 h. Wells were washed with PBS twice after incubation to remove unbound Hoechst dye and analyzed under a microscope (Olympus FV 3000) for counting the stained nuclei. Finally, the cell counting was performed manually with the help of images obtained from the microscope.

### In vitro cell migration assay

For measurement of cell motility, 2D cell migration assay from (Marwitz et al., 2016) was used. Cells were seeded in 24-well plate (Zell Kontakt 3231-20) at a density of 2,000–5,000 cells/well (depends from cell lines). Twenty-four hours after seeding the cells were treated with various concentrations of Withaferin-A and Silibinin for a total of 24 h and after stained with Hoechst (Sigma) for 1 h. Images were acquired in 20 min intervals for 48 h using environment-controlled microscope (IX81, Olympus) equipped with an UPlanSApo 10 ×/0.4 objective lens (Olympus). Nine positions per well (3 ×3 grid) were imaged and stitched with a ImageJ plugin (Preibisch, Saalfeld & Tomancak, 2009). Single cell tracking was performed with ImageJ Mtrack2 plugin. Speed of each tracked cell was calculated by dividing total travelled distance by total time, for which cell was tracked. Persistence was calculated by dividing the distance between the first and the last point, where the cell was tracked, by total travelled distance. Resulting number was multiplied by the square root of time, for which cell was tracked divided by maximal possible tracking time, in order to penalize cells, which were tracked for a shorter period of time.

### Immunofluorescence and microscopic imaging

Cells were fixed for 5 min in 3.7% PFA in PBS at RT, permeabilized with ice-cold 0.1% TritonX-100 in PBS for 5 min, blocked for 30 min with Blocking solution (3% BSA, 2.5% FCS) and stained with primary antibodies to vimentin and actin (Abcam, Cambridge, UK) for 1 h. After staying cells were washed 3 times in PBS and stained with secondary antibodies, Phalloidin-BODIBY (Thermo Fisher) and Hoechst (Sigma) for 30 min. Imaging was performed using the confocal laser scanning microscope (Olympus FV 3000) with 64x oil immersion lens to visualize the actin and vimentin network as well as the nucleus in the cells.

### Gene expression data

Gene expression (GE) data of H1650, H1975, A549, H838, H2030 cell lines from GSE47206 (El-Chaar et al., 2014) were used for computational analysis of correlation between transcriptomic and IC50 profiles of NSCLC cells. Analysis was performed only for transcripts with the detection significance of *p* < 0.05 (P,M) in all five NSCLC cell lines. Trancripts with the non-significant level of detection (A) were excluded from analysis.

### GO enrichment analysis

Gene ontology enrichment analysis was performed using STRING v11 (Szklarczyk et al., 2019) with the significance level of *p* < 0.05 after multiple testing correction (Benyamini & Hochberg, 1995) for each functional classification framework (GO, KEGG, InterPro, etc.).

### Transfection of NSCLC cells

All siRNA used for knockdown of *BIRC5*, *FOXM1*, *BRCA1* was purchased from Ambion^®^ThermoFisher Scientific, see Table 3.

**Table 3:** List of siRNAs used for protein knockdown by gene silencing.

Gene	Full Name	Sense siRNA Sequence	Antisense siRNA Sequence
BIRC5	Baculoviral IAP repeat containing 5	GGACCACCGCAUCUCUACATT	UGUAGAGAUGCGGUGGUCCTT
BIRC5	Baculoviral IAP repeat containing 5	GCAGGUUCCUUAUCUGUCATT	UGACAGAUAAGGAACCUGCAG
BIRC5	Baculoviral IAP repeat containing 5	CAAAGGAAACCAACAAUAATT	UUAUUGUUGGUUUCCUUUGCA
FOXM1	Forkhead box M1	GCUCAUACCUGGUACCUAUTT	AUAGGUACCAGGUAUGAGCTG
FOXM1	Forkhead box M1	CACUAUCAACAAUAGCCUATT	UAGGCUAUUGUUGAUAGUGCA
FOXM1	Forkhead box M1	GGAUCAAGAUUAUUAACCATT	UGGUUAAUAAUCUUGAUCCCA
BRCA1	Breast cancer 1, early onset	GGGAUACCAUGCAACAUAATT	UUAUGUUGCAUGGUAUCCCTC
BRCA1	Breast cancer 1, early onset	CAGCUACCCUUCCAUCAUATT	UAUGAUGGAAGGGUAGCUGTT
BRCA1	Breast cancer 1, early onset	CAUGCAACAUAACCUGAUATT	UAUCAGGUUAUGUUGCAUGGT

Transfection experiments were carried out by lipofection technique using Lipofectamine^®^2000 (Invitrogen), with transient expression. Two types of transfection methods were performed in the optimization of transfection efficiency: reverse and forward transfection.

### Forward and reverse transfection method

Forward transfection is a conventional method of transfection where cells are first seeded for 24 h and stored at 37 °C and 5% CO_2_. After incubation, cells are transfected as per company’s (Invitrogen) protocol. In reverse transfection, unlike conventional transfection, the genetic material is coated on the bottom of well plates prior to the seeding of NSCLC cells into multi-well plate. As the order of adding genetic material is reverse compared to the conventional method it is named as reverse transfection method. Multi-well plates are coated with required siRNA to be transfected along with gelatin, fibronectin and transfection reagent (Lipofectamine^®^2000) stored at 4 °C for 28 h and stored in dry place. Later, NSCLC cells were seeded into these wells, incubated for 28 h to attain transfected cells.

### Detection of siRNA mediated gene silencing

Estimation of transfection efficiency of the NSCLC cells was performed by blocking PLK1 (polo-like-kinase-1) enzyme using RNA silencing technique via small interfering RNA (siRNA). Expression of PLK1 has several functions like the initiation of mitosis, cytoplasmic separation, mitotic spindle formation and membrane formation in telophase of mitosis by phosphorylating mitotic kinesin-like protein-1. Silencing of PLK1 leads to mitotic cell arrest and the centrosomes lose the ability to nucleate microtubules. This effect can be observed under a microscope with star shape nuclear phenotype. Presence of star shape nucleus indicates that the PLK1 was successfully silenced that in turn confirms that the cells are transfectable.

### Immunoblotting

The confirmation of knockdown efficiency was carried out using Western Blot analysis. Harvesting and samples for analysis were prepared by removing supernatant from the cells followed by addition of 500 µl of PBS. Cells were resuspended and transferred to 1.5 ml for centrifugation for 5 min at 1,600 RPM. After centrifugation, PBS was removed without disturbing cell pellet. 25 µl of 1x Laemlli buffer containing DTT (final concentration 100 mM) was added and incubated for 10 min at 95 °C. Proteins in the sample were separated by SDS-PAGE in a 10% resolving gel and transferred onto methanol-activated PVDF membranes by semi-dry blotting technique using transfer buffer at a constant current of 130 V until the sample reaches the resolving gel. As soon as the sample reaches resolving gel electrophoresis is run at 90V. The membrane was blocked with 5% milk powder, 0.5% BSA in PBST for 1 h at room temperature. After blocking, the membrane was incubated with specific primary antibody in 5% (w/v) milk powder/PBST overnight at 4 °C. To remove the unbound primary antibody three subsequent washings for 10 min with PBST were performed. Then the membrane was incubated with the horseradish peroxidase (HRP) coupled secondary antibody in 5% (w/v) milk powder, 0.5% BSA in PBST for 1 h at room temperature. Three subsequent PBST washing were performed to ensure complete removal of the secondary antibody. Detection of proteins was carried out by using ECL chemiluminescent immunodetection system with different exposure time using INTAS Fluoreszenz u. ECL Imager. Subsequently, quantitative analysis of Western Blot images was performed using ImageJ according to a standard protocol (http://www.openwetware.org/wiki/Protein_Quantification_Using_ImageJ). Figure S5 shows exemplary analysis of transient knockdown of target proteins *FOXM1* and *BIRC5* in A549 and H838 NSCLC cells using small-interfering RNA (siRNA) vs *β*-actin as a loading control.

**Figure 1:**
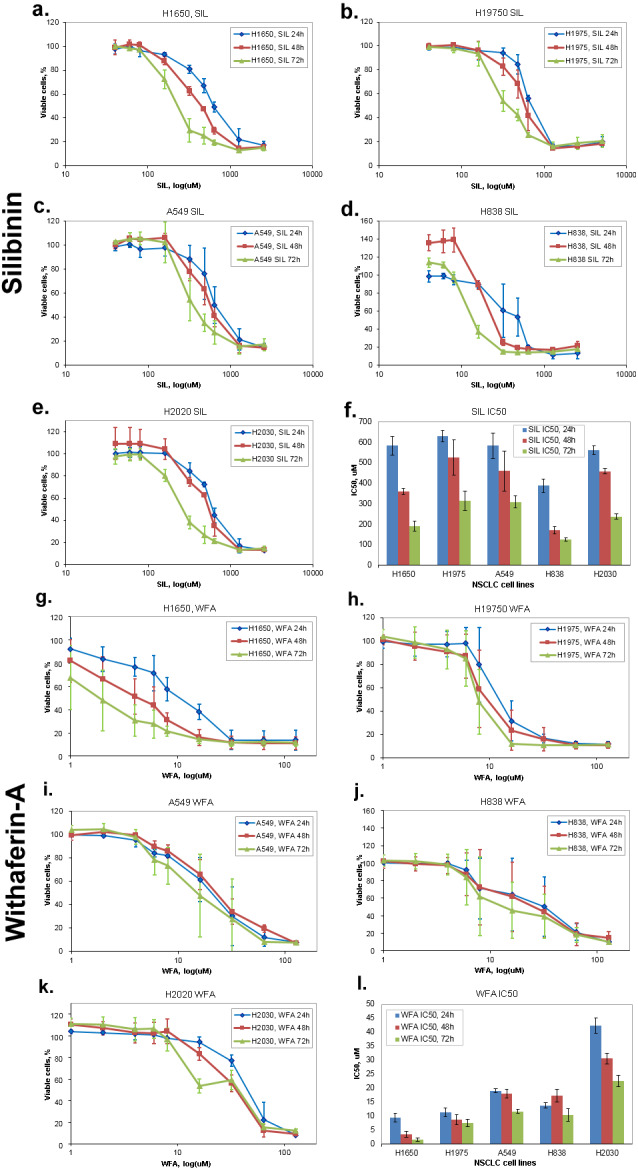
Dose-dependent 24 h, 48 h, 72 h response of NSCLC cells lines to Silibinin (A–F) and Withaferin-A (G–L): (A, G) H1650, (B, H) H1975, (C, I) A549, (D, J) H838, (E, K) H2030, (F, L) mean SIL IC50 values for all five NSCLC cell lines. Error bars indicate stdev of measurements performed with three replicates.

## Results

The intrinsic EMT stage of NSCLC cell lines was ranked according to the expression level of E-cadherin (*CDH1*) as suggested by Byers et al. (2013), see Table S1. Accordingly, five NSCLC cell lines (H1650, H1975, A549, H838, H2030) exhibit gradual difference in their intrinsic EMT stage: from most epithelial (H1650) to most mesenchymal (H2030), respectively.

Dose-dependent response of all five NSCLC cell lines to 24 h, 48 h and 72 h exposure with Silibinin (SIL) and the reference compound, Withaferin-A (WFA), measured via the CellTiter-Blue^^®^^ Cell Viability Assay was used to determine IC50 values of both compounds. Figure 1 shows dose response curves and SIL/WFA IC50 values of all five NSCLC cell lines.

**Figure 2:**
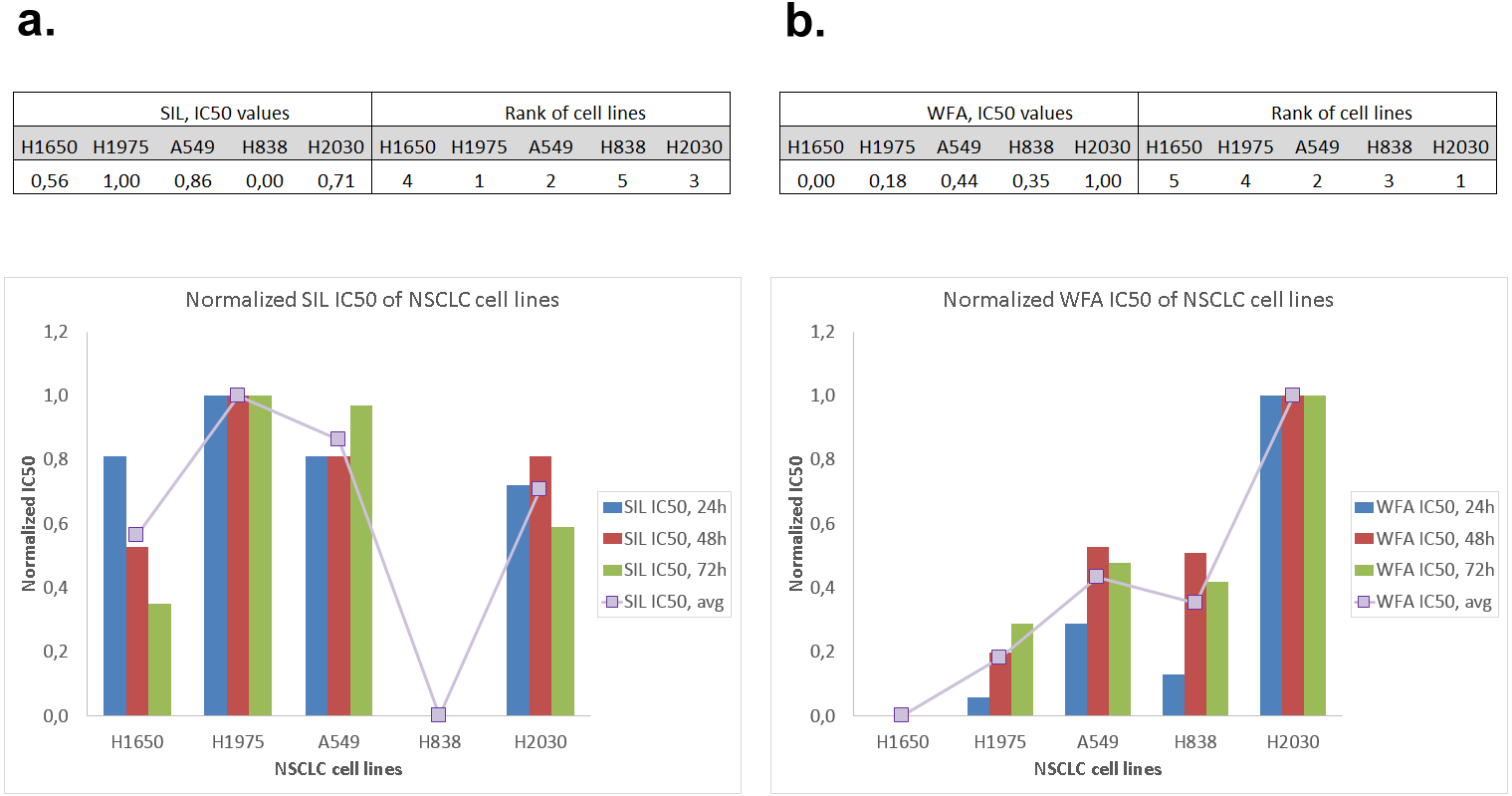
Normalized 24 h, 48 h, 72 h and average IC50 of five NSCLC cell lines: (A) Silibinin, (B) Withaferin-A.

As one can see from the overview of SIL/WFA IC50 ranks of all five NSCLC cell lines in Fig. 2, the pattern of WFA IC50 values exhibits a strong correlation with the intrinsic EMT stage of NSCLC cells, while SIL IC50 does not show such a correlation. Strikingly, all cell lines with exception of H838 have a comparable level of SIL IC50.

**Figure 3:**
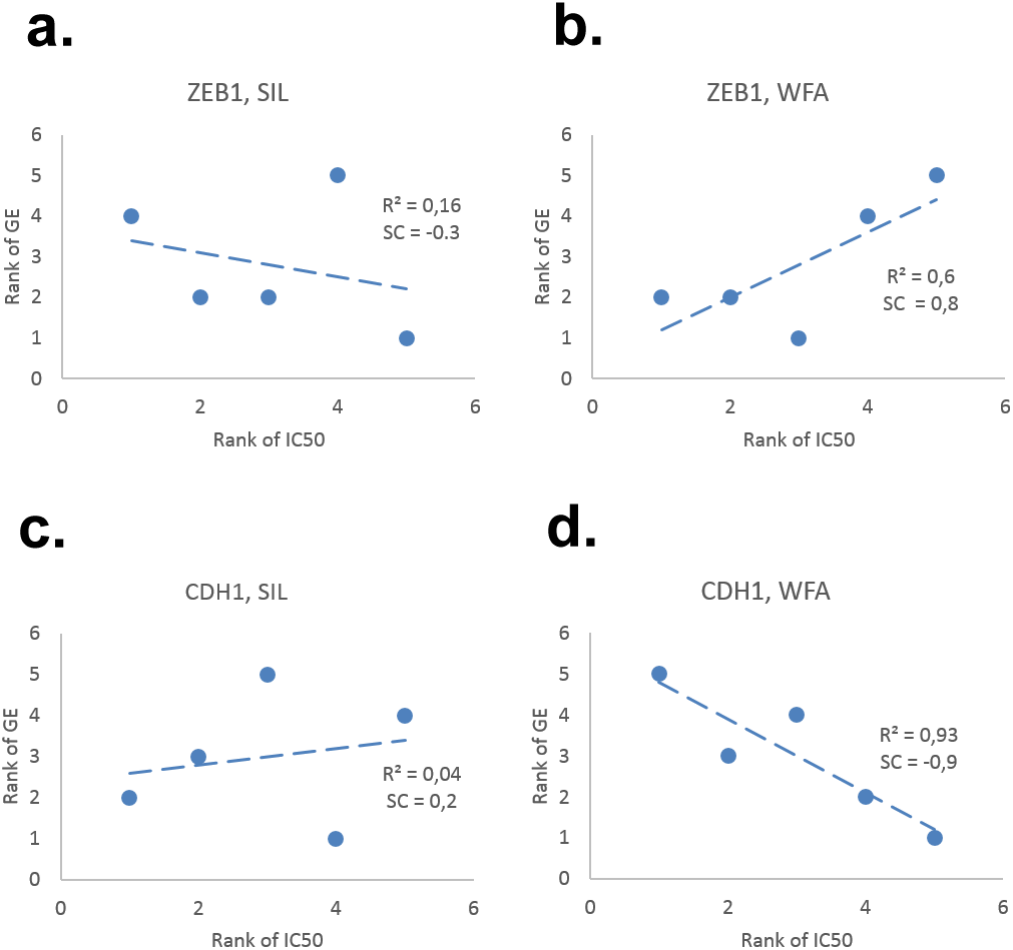
Examples of correlation between expression of (A, B) mesenchymal (*ZEB1*) and (C,D) epithelial (*CDH1*) genes with SIL/WFA IC50 in five NSCLC cell lines. While WFA IC50 strongly correlates with the pattern of EMT gene expression in NSCLC cells, SIL IC50 does not show any significant correlation with EMT genes; see details in Table S1.

To test possible dependency of the drug response on the intrinsic EMT stage of NSCLC cells, Spearman (rank) correlation between the pattern of normalized SIL and WFA IC50 and the EMT 58 gene signature from Byers et al. (2013) was calculated. From analysis of correlations between expression of 58 EMT genes and the pattern of SIL/WFA IC50 response, it follows that the WFA IC50 of NSCLC cells exhibits a significantly higher correlation with the EMT stage than the SIL IC50 response, see Table S1. Examples of significant WFA and poor SIL correlations with two major biomarkers of EMT (e.g., *CDH1* and *ZEB1*) are shown in Fig. 3. While gradient of WFA IC50 values from epithelial to mesenchymal cell lines is consistent with previous observations of elevated drug-resistance and survival potential of mesenchymal cells (Tan et al., 2014), low correlation of SIL IC50 with EMT gene expression (GE) appear to contradict this prevailing view.

**Figure 4:**
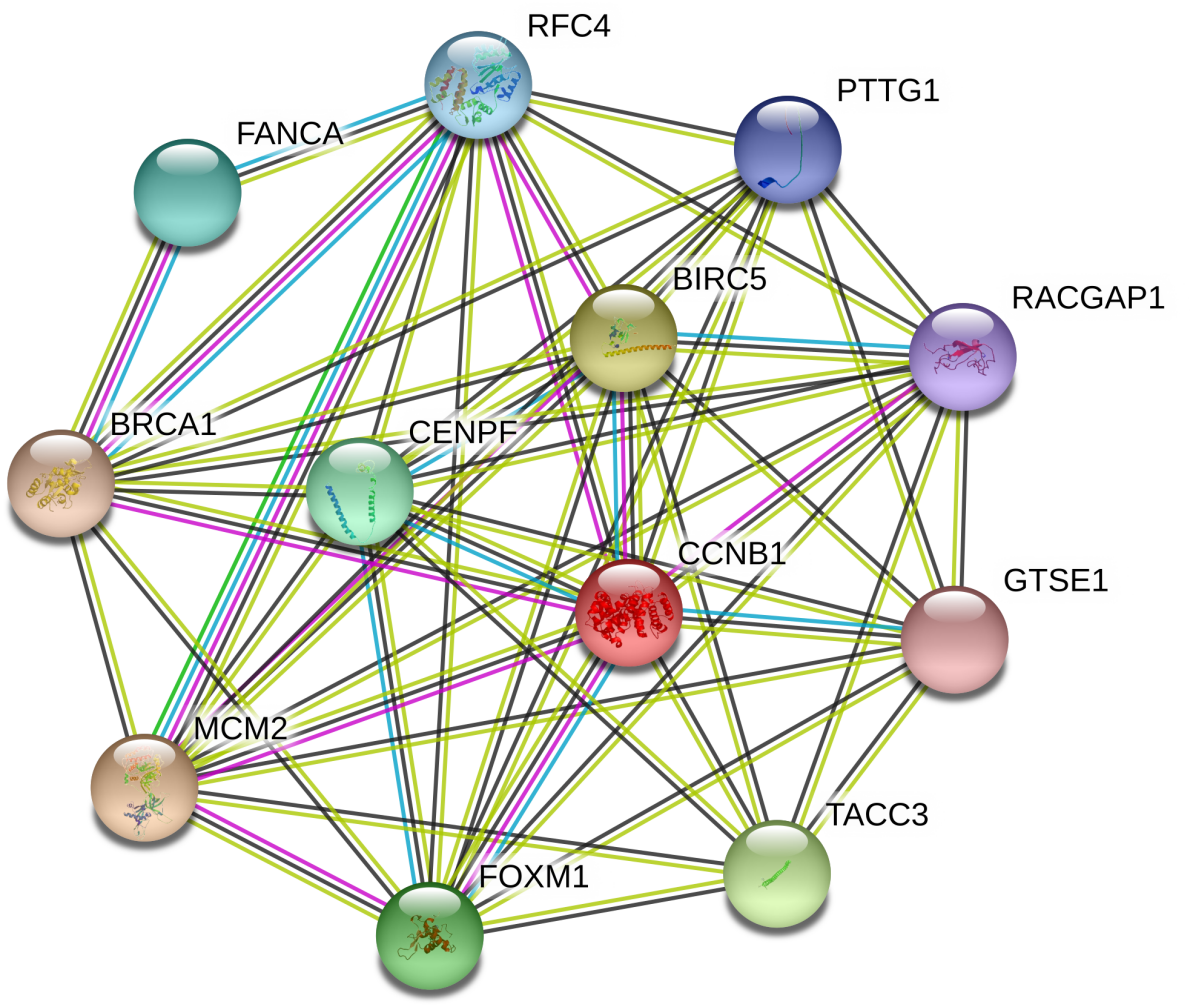
Visualization of a subnetwork of tightly interconnected 12 genes from the overlap between the groups of 144 SIL-response relevant genes in five NSCLC cell lines and 90 high-communicability pan-cancer genes from (Gladilin & Eils, 2017) including three prominent targets of the *STAT3* transcription factor: *BIRC5*, *FOXM1*, *BRCA1* using STRING v11 (Szklarczyk et al., 2019) with default settings.

**Figure 5:**
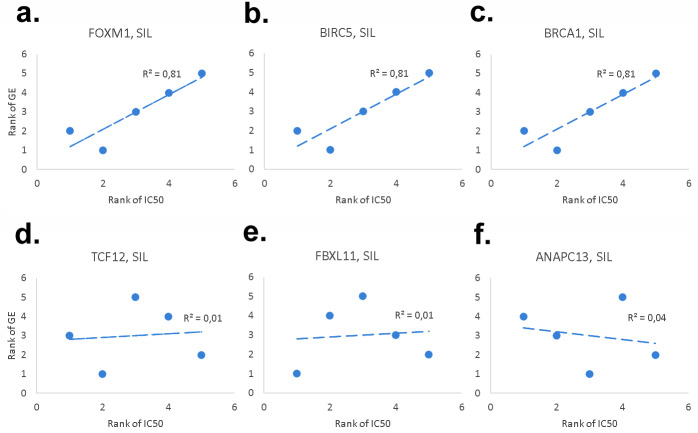
Examples of genes with significant (*FOXM1* (A), *BIRC5* (B), *BRCA1* (C)) and non-significant (*TCF12* (D), *FBXL11* (E), *ANAPC13* (F)) correlation between the patterns of gene expression and normalized SIL IC50 in five NSCLC cell lines.

In order to dissect further possible genes correlating with the pattern of SIL IC50 among five NSCLC cell lines, correlation analysis was performed at the whole genome scale under consideration of reliability of transcriptomic signals (i.e., M/P accepted, A excluded), see Table S2. From this analysis 144 genes with a positive correlation and significance level of *p* < 0.05 were identified. Gene ontology (GO) analysis of 144 genes positively correlating with the SIL IC50 signature of five NSCLC cell lines shows a significant enrichment in GO categories related to cell cycle, G2/M transition, nuclear localization, DNA-replication and repair, and related signaling and metabolic processes and functions, see Table S2. Furthermore, a statistically significant overlap between this group of 144 genes and 90 ‘high-communicability’ genes from our previous pan-cancer analysis study (Gladilin & Eils, 2017) was detected (12/90=13.3 %, *p* < 0.001 hypergeometric test: hgt(22277, 144, 90, 12) = 5.7e−13), see Table S3. Notably, this set of 12 cell cycle process related genes includes three genes that are known to be prominent targets of *STAT3* transcription factor: *BIRC5* (Alvarez & Frank, 2004; Gritsko et al., 2006; Carpenter & Lo, 2014), *FOXM1* (Mencalha et al., 2012), *BRCA1* (Snyder, Huang & Zhang, 2007), see Fig. 4. Figure 5 shows correlation between gene expression patterns of these three *STAT3* target genes and SIL IC50 response of five NSCLC cells vs. another three non-significantly correlating genes.

To evaluate the relevancy of computationally selected genes to SIL viability response of NSCLC cells, transient knockdown of *BIRC5*, *FOXM1*, *BRCA1* genes was performed. Even though the reverse transfection method was considered to be more efficient providing higher transfection rates with minimal nucleic acid usage (Erfle et al., 2007), here we found out forward transfection to show a better performance crossover five tested NSCLC cell lines, see Fig. S1. Reverse and forward transfection of siRNA targeting PLK1 resulted in the comparable phenotype frequencies in all tested cell lines. A459 and H838 responded strongly in both methods, whereas, H1650 and H2030 showed no phenotype. This may be attributed either to difficulties in transfecting these cells or non-responsiveness to PLK1 depletion. Nearly 50% of H1975 cells showed prometaphase arrest when using forward transfection, but hardly any effect was observed under the conditions of reverse transfection. Consequently, the forward transfection protocol was used for transfection of H1975, A549, H838 cells with target siRNA.

**Figure 6:**
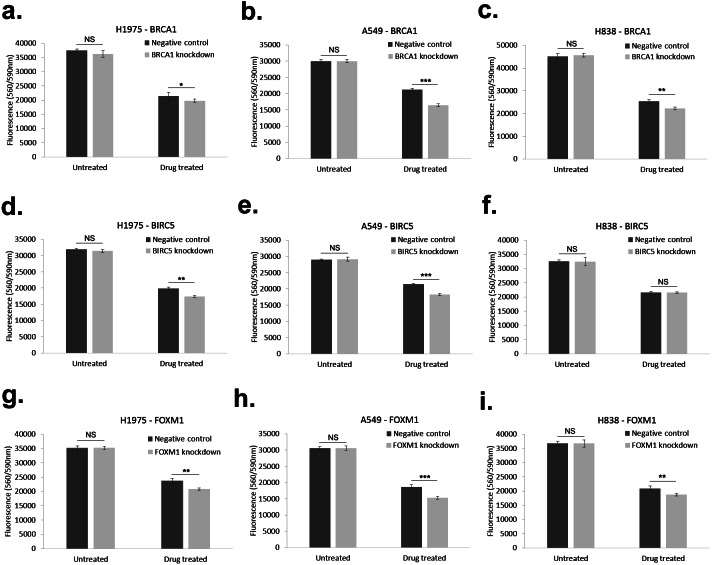
Summary of cell viability measurements of BRCA1 (A–C), BIRC5 (D–F) and FOXM1 (G–I) knocked down NSCLC cell lines with and without Silibinin treatment of H1975 (A, D, G), A549 (B, E, H) and H838 (C, F, I) NSCLC cell lines (see Table S4 for raw data for these plots). Error bars indicate stdev of measurements performed with three replicates. Negative control = NSCLC cells + lipofectamine^®^2000 + scramble siRNA, knockdown = NSCLC cells + lipofectamine^®^2000 + siRNA. NS, *p* > 0.05 (non-significant); *, *p* < 0.05; **, *p* < 0.01; ***, *p* < 0.001.

Comparative viability measurements of H1975, A549, H838 cells with transiently knocked down *BIRC5*, *FOXM1*, *BRCA1* genes confirmed their relevancy to SIL response with exception of *BIRC5* knockdown in H838 cells, see Fig. 6. We draw the negative result of *BIRC5* knockdown in H838 cells back to constitutively low level of *BIRC5* in H838 in comparison to H1975, A549 cell lines. The relative changes in SIL response between control and knocked down NSCNC cells observed in this work are in the order of magnitude comparable with previous observations for other small weight compounds and NSCLC cell lines (Gao et al., 2020).

## Discussion

**Figure 7:**
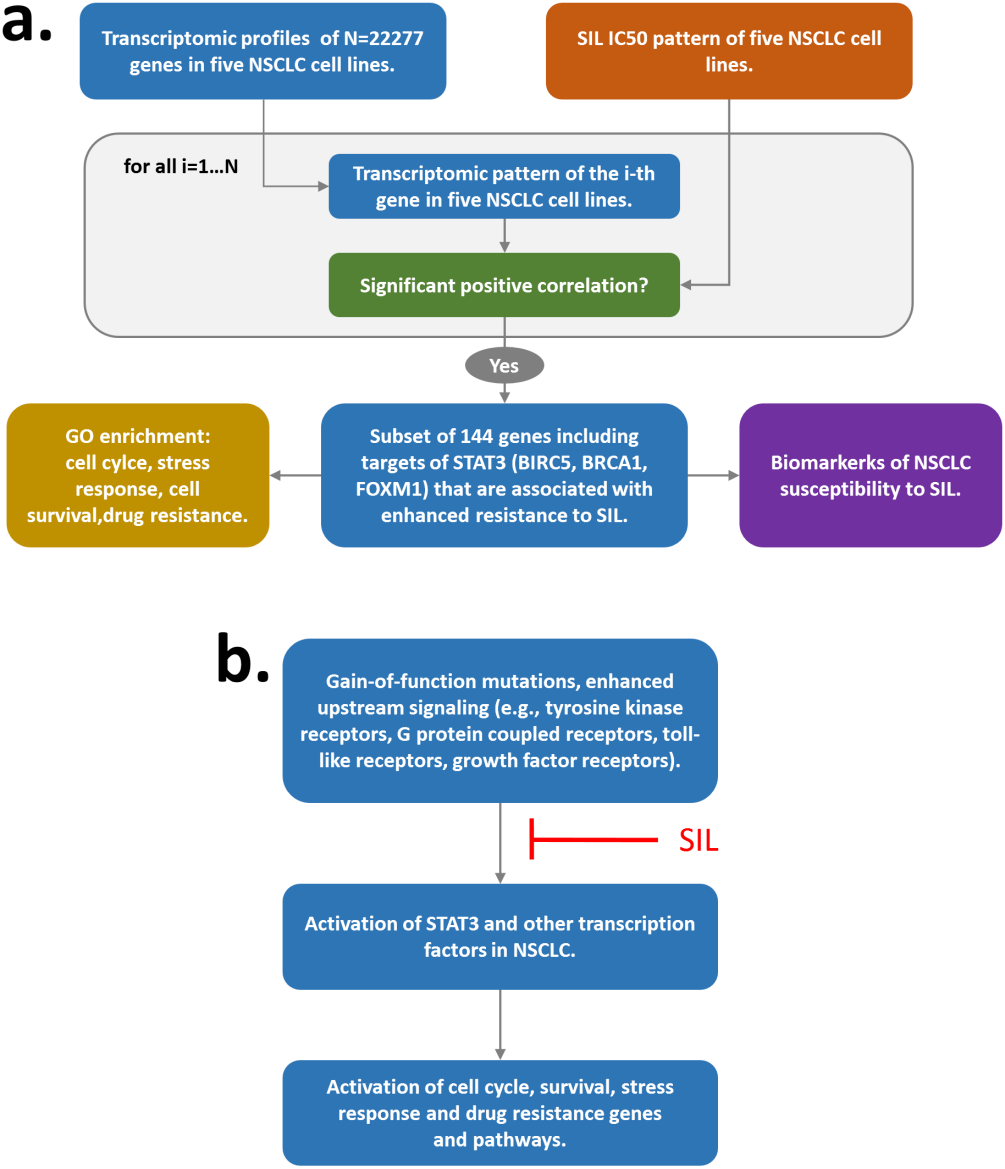
Overall scheme of the study (A) and mechanistic explanation of experimental results (B).

There is abundant evidence that Silibinin exhibits remarkable antineoplastic effects crossover different cancer tissue/cell types by affecting multiple molecular targets and pathways. However, not much is known about factors responsible for individual SIL viability response of cancer cells. Here, we systematically analyzed the relationship between the patterns of SIL IC50 and gene expression in five NSCLC cell lines exhibiting gradual difference with respect to their intrinsic EMT stage and compared it with the reference compound Withaferin-A. Our experimental results showed that, differently from WFA and other drugs, sensitivity of NSCLC cells to Silibinin does not correlate with the intrinsic EMT stage. Instead, a subset of cell cycle, survival and stress responsive genes including three prominent targets of *STAT3* (i.e., *BIRC5*, *FOXM1*, *BRCA1*) was found to exhibit significant correlation with the pattern of SIL IC50 in five NSCLC cell lines, see Fig. 7A. Subsequent evaluation of SIL viability response of transiently *BIRC5*, *FOXM1*, *BRCA1* silenced NSCLC cells confirmed computationally predicted dependency of SIL dose on the expression level of these genes. Since the expression of *STAT3* transcriptional targets but not *STAT3* itself significantly correlates with SIL IC50 of five NSCLC cell lines, we conclude that external and/or constitutive activation of *STAT3*, for example, due to gain-of-function mutations or enhanced upstream signaling (e.g., tyrosine kinase receptors, G protein coupled receptors, toll-like receptors, growth factor receptors) is responsible for differences in SIL sensitivity between different NSCLC cell lines, see Fig. 7B. In this point, our study confirms previous findings that inhibition *STAT3* and its downstream targets is one of the major mechanisms of Silibin antineoplastic action. Furthermore, our observations confirm previous findings that expression of known drug targets does not necessarily exhibit a significant correlation with IC50 (Parca et al., 2019), and that downstream targets of those drug targets may instead represent a more reliable reference for IC50 correlation studies.

Our correlation analysis between viability and whole trascriptome profiles of five NSCLC cell lines provides a rich source of information for further investigation of anti-neoplastic and EMT-inhibiting effects of Silibinin in cancer cells. Among the group of 144 proteins whose expression positively correlates with the SIL IC50 there are also significantly enriched ontological categories related to cytoskeleton regulation and organization, see Table S2. Our previous studies (Gladilin, 2015) indicated remarkable inhibiting effects of Silibinin on cancer cell migration and overall reorganization of vimentin and actin networks (Supplementary Figs. S2 and S3) that can be attributed to these cytoskeleton-related genes, see Fig. S4. While GO enrichment in this work was performed using conventional statistical testing, rapidly evolving deep learning techniques (e.g., Cao et al., 2018; Le et al., 2019) hold the promise of proving more in-depth insights into large gene expression and cell viability data in the future.

Furthermore, our experimental results suggest that the intrinsic EMT stage is not a suitable biomarker of SIL dose response of cancer cells. Instead, the subset of SIL IC50 correlating genes determined in this work can be used for dose optimization in adjuvant tumor therapy using Silibinin. Further investigations with a broader spectrum of NSCLC cells and other cancer cell lines are required to generalize preliminary findings of this study.

## Conclusion

Silibinin inhibits NSCLC cell viability and motility in a dose-dependent manner. Thereby, SIL IC50 of different NSCLC cells does not correlate with their transcriptomic EMT signature. Instead, it was found that SIL dose–response correlates with the expression level of a network of 144 cell cycles, stress responsive and survival proteins including some well known targets of *STAT3*. It was shown that selected downstream targets of *STAT3* including *BRCA1*, *BIRC5*, *FOXM1* have impact on Silibinin dose response.

##  Supplemental Information

10.7717/peerj.10373/supp-1 Supplemental Information 1 Raw CTB IC50 data and ranking of NSCLC cell lines according to IC50 vs EMT Raw data of CTB IC50 measurements of five NSCLC cell lines (H1650, H1975, A549, H838, H2030) as well as their ranking according to the EMT gene signature from Byers et al 2013 and its correlation with the pattern of normalized SIL/WFA IC50 values.

10.7717/peerj.10373/supp-2 Supplemental Information 2 Genome wide correlation between patterns of gene expression and SIL IC50 response in five NSCLC cell lines (H1650, H1975, A549, H838, H2030) GO enrichment terms of 144 positively and 281 negative correlating genes.

10.7717/peerj.10373/supp-3 Supplemental Information 3 SIL-response correlating genes Overlap between the set of 144 positively SIL-response correlating genes (from Supplementary Table S2) and 90 pan-cancer genes from Gladilin & Eils 2017 including three prominent targets of STAT3 transcription factor: BIRC5, FOXM1, BRCA1.

10.7717/peerj.10373/supp-4 Supplemental Information 4 Raw data of cell viability measurements of knocked down NSCLC cells vs untreated control Raw data of cell viability measurements of BRCA1, BIRC5 and FOXM1 knocked down NSCLC cell lines with and without Silibinin treatment of H1975, A549 and H838 NSCLC cell lines.

10.7717/peerj.10373/supp-5 Supplemental Information 5 Tests of efficiency of reverse (top) and forward (bottom) transfection protocols in NSCLC cell lines A. Star shape nuclear phenotypes formed due to mitotic arrest by PLK1 siRNA transfection using solid phase reverse and forward transfections in five NSCLC cell lines as described. PLK1 was used as a reference siRNA to measure the transfectability of NSCLC cell lines. B. Phenotype incidence of PLK1 siRNA transfected NSCLC cells using solid-phase reverse and forward transfections derived from three technical and three biological independent replicates; error bars represents standard deviations.

10.7717/peerj.10373/supp-6 Supplemental Information 6 Cell motility measurements of drug treated H1975 cells Dose-dependent effects of (a) Silibinin and (b) Withaferin-A on 2D migration of H1975 cells.

10.7717/peerj.10373/supp-7 Supplemental Information 7 Microscopic imaging of actin, vimentin in drug treated H1975 cells CLSM imaging of effects of Withaferin-A and Silibinin treatment vs untreated control on vimentin (red) and actin (green) in H1975 cells.

10.7717/peerj.10373/supp-8 Supplemental Information 8 Subnetwork of cytoskeleton-related genes affected by Silibinin Visualization of a subnetwork of 17 cytoskeleton-related genes whose expression positively correlates with the pattern of SIL IC50 in five NSCLC cells using STRING v11.

10.7717/peerj.10373/supp-9 Supplemental Information 9 Exemplary analysis of transient knockdown of FOXM1 and BIRC5 proteins using siRNA in two NSCLC cell lines Western blot images representing the knockdown of FOXM1 and BIRC5 in (A) A549 and (B) H838 cell lines using small-interfering RNA (siRNA) knockdown vs *β*-actin as a loading control. The Western Blot mesaurements were performed twice resulting in similar observations. WT denote measurements in cells from the master cell culture without siRNA and NC are measurements in cells used in the experiment without siRNA.

## Additional Information and Declarations

### Data Availability

The following information was supplied regarding data availability: The raw data are available in Tables S1–S4.
