# Supplementary figures and images for: Transcriptome profiling reveals Silibinin dose-dependent response network in non-small lung cancer cells
Source: PeerJ. 2020 Dec 16;8:e10373. doi: 10.7717/peerj.10373 (PMC7749657; doi:10.7717/peerj.10373)

## Reverse transfection

A.

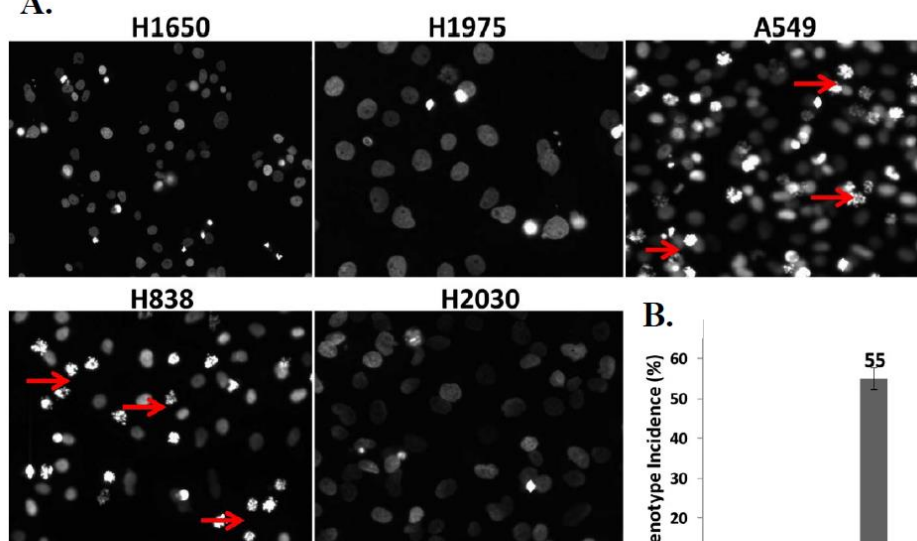

B.

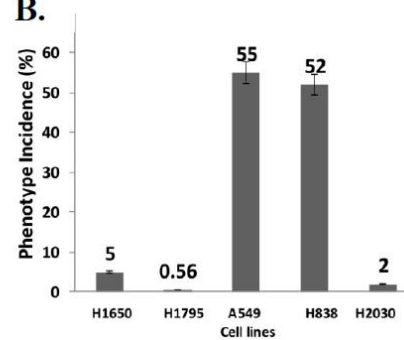

## Forward transfection

A.

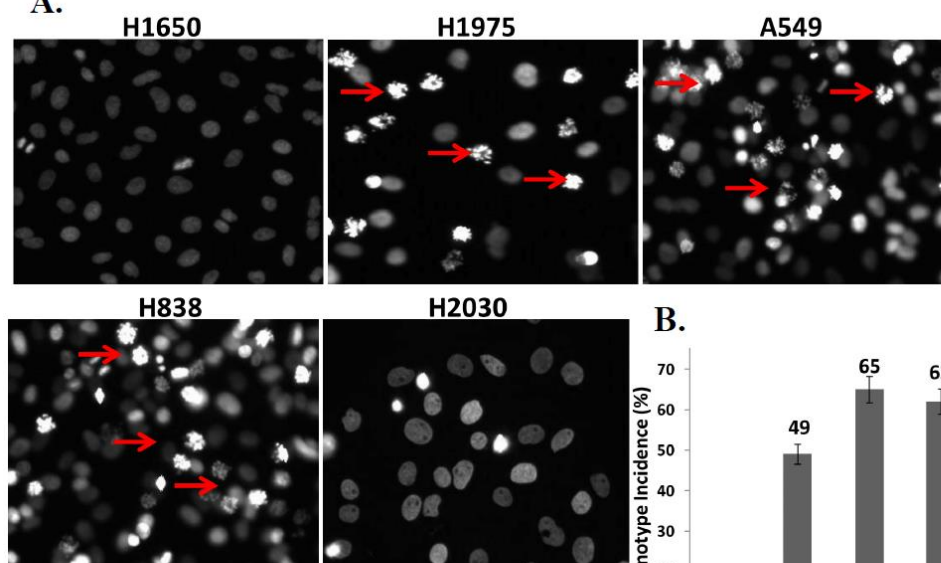

B.

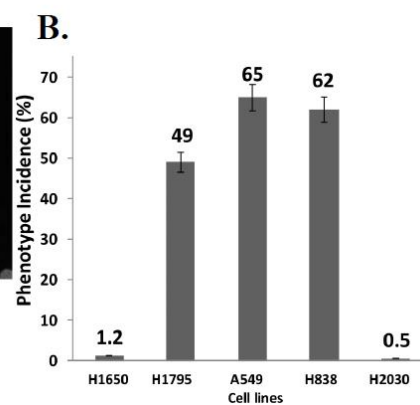

Supplement: Supplemental Information 5 — A. Star shape nuclear phenotypes formed due to mitotic arrest by PLK1 siRNA transfection using solid phase reverse and forward transfections in five NSCLC cell lines as described. PLK1 was used as a reference siRNA to measure the transfectability of NSCLC cell lines. B. Phenotype incidence of PLK1 siRNA transfected NSCLC cells using solid-phase reverse and forward transfections derived from three technical and three biological independent replicates; error bars represents standard deviations. [file peerj-08-10373-s005.pdf]

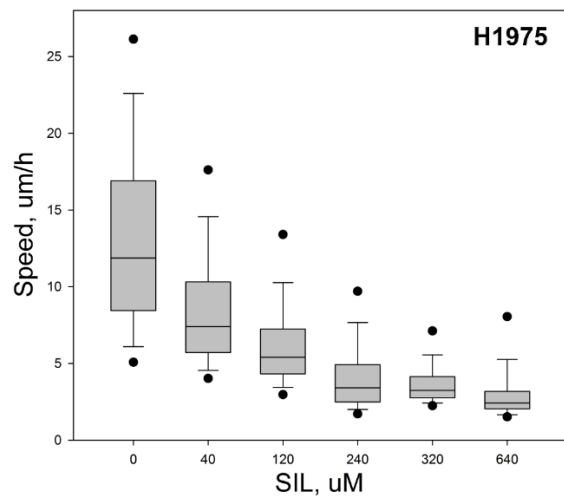

**(a)**

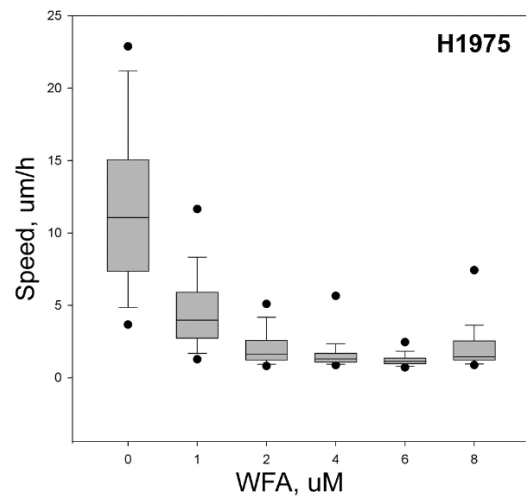

**(b)**

Supplement: Supplemental Information 6 — Dose-dependent effects of (a) Silibinin and (b) Withaferin-A on 2D migration of H1975 cells. [file peerj-08-10373-s006.pdf]

H1975, untreated

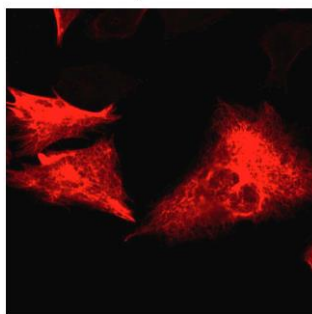

H1975, WFA 6uM

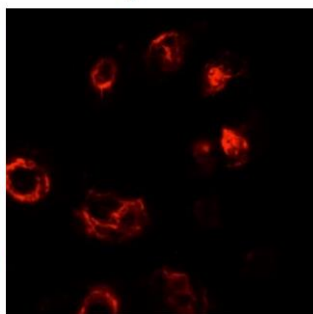

H1975, SIL 640 uM

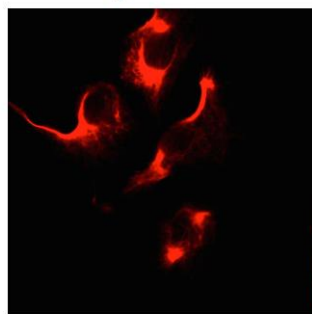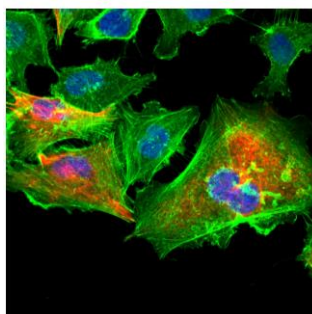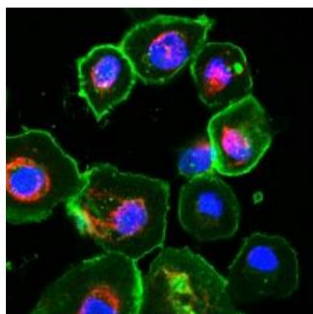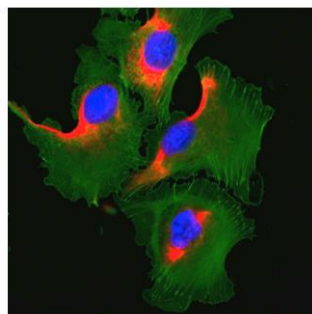

Supplement: Supplemental Information 7 — CLSM imaging of effects of Withaferin-A and Silibinin treatment vs untreated control on vimentin (red) and actin (green) in H1975 cells. [file peerj-08-10373-s007.pdf]

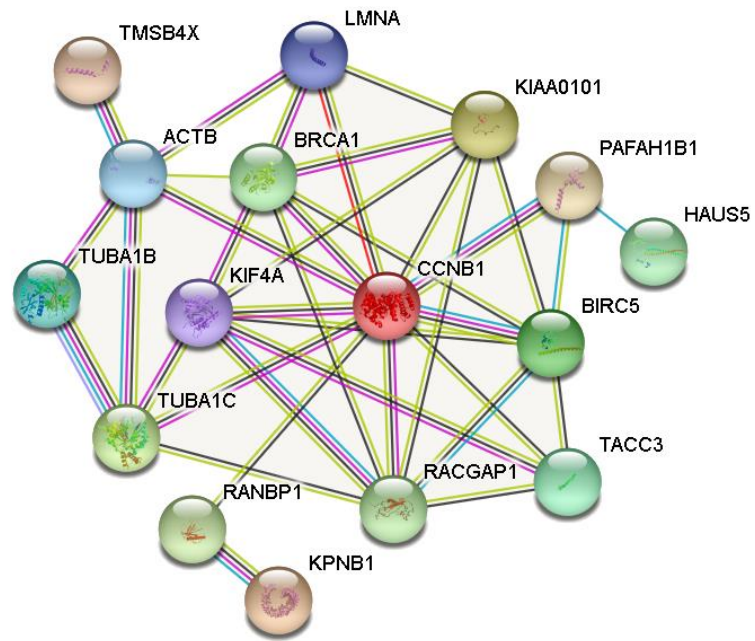

Supplement: Supplemental Information 8 — Visualization of a subnetwork of 17 cytoskeleton-related genes whose expression positively correlates with the pattern of SIL IC50 in five NSCLC cells using STRING v11. [file peerj-08-10373-s008.pdf]

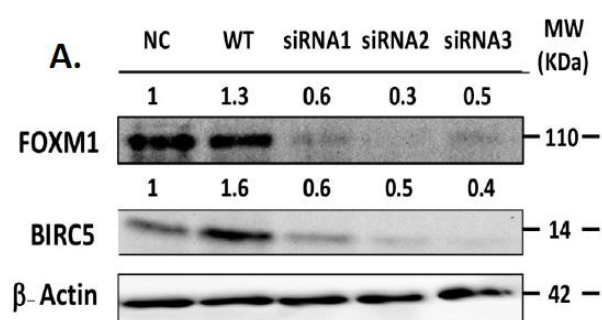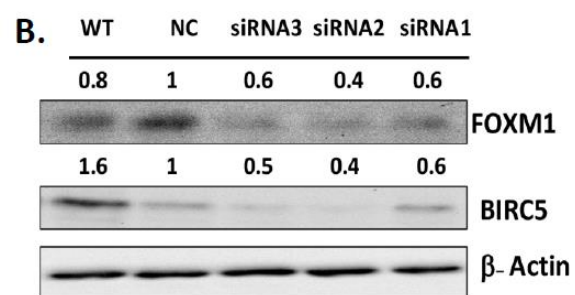

Supplement: Supplemental Information 9 — Western blot images representing the knockdown of FOXM1 and BIRC5 in (A) A549 and (B) H838 cell lines using small-interfering RNA (siRNA) knockdown vs β-actin as a loading control. The Western Blot mesaurements were performed twice resulting in similar observations. WT denote measurements in cells from the master cell culture without siRNA and NC are measurements in cells used in the experiment without siRNA. [file peerj-08-10373-s009.pdf]
